# Transforming growth factor-β1 induces EMT by the transactivation of epidermal growth factor signaling through HA/CD44 in lung and breast cancer cells

**DOI:** 10.3892/ijmm.2015.2222

**Published:** 2015-05-25

**Authors:** LINGMEI LI, LISHA QI, ZHIJIE LIANG, WANGZHAO SONG, YANXUE LIU, YALEI WANG, BAOCUN SUN, BIN ZHANG, WENFENG CAO

**Affiliations:** 1 Department of Pathology, Tianjin Medical University Cancer Institute and Hospital, Tianjin 300060, P.R. China; 2 Department of Breast Cancer, Tianjin Medical University Cancer Institute and Hospital, Tianjin 300060, P.R. China; 3 Tianjin Medical University, Tianjin 300070, China; Tianjin Medical University, Ministry of Education, Tianjin 300060, P.R. China; 4 National Clinical Research Center for Cancer; Tianjin Medical University, Ministry of Education, Tianjin 300060, P.R. China; 5 The Key Laboratory of Cancer Prevention and Therapy; Tianjin Medical University, Ministry of Education, Tianjin 300060, P.R. China; 6 Key Laboratory of Breast Cancer Prevention and Therapy, Tianjin Medical University, Ministry of Education, Tianjin 300060, P.R. China

**Keywords:** epithelial-mesenchymal transition, transforming growth factor-β1, hyaluronan, CD44, epidermal growth factor

## Abstract

Epithelial-mesenchymal transition (EMT), a process closely related to tumor development, is regulated by a variety of signaling pathways and growth factors, such as transforming growth factor-β1 (TGF-β1) and epidermal growth factor (EGF). Hyaluronan (HA) has been shown to induce EMT through either TGF-β1 or EGF signaling and to be a regulator of the crosstalk between these two pathways in fibroblasts. In this study, in order to clarify whether HA has the same effect in tumor cells, we utilized the lung cancer cell line, A549, and the breast cancer cell line, MCF-7, and found that the effects of stimulation with TGF-β1 were more potent than those of EGF in regulating the expression of EMT-associated proteins and in enhancing cell migration and invasion. In addition, we observed that TGF-β1 activated EGF receptor (EGFR) and its downstream AKT and extracellular signal-regulated kinase (ERK) pathways. Furthermore, we found that TGF-β1 upregulated the expression of hyaluronan synthases (HAS1, HAS2 and HAS3) and promoted the expression of CD44, a cell surface receptor for HA, which interacts with EGFR, resulting in the activation of the downstream AKT and ERK pathways. Conversely, treatment with 4-methylumbelliferone (4-MU; an inhibitor of HAS) prior to stimulation with TGF-β1, inhibited the expression of CD44 and EGFR, abolished the interaction between CD44 and EGFR. Furthermore, the use of shRNA targeting CD44 impaired the expression of EGFR, deactivated the AKT and ERK pathways, reversed EMT and decreased the migration and invasion ability of cells. In conclusion, our data demonstrate that TGF-β1 induces EMT by the transactivation of EGF signaling through HA/CD44 in lung and breast cancer cells.

## Introduction

Epithelial-mesenchymal transition (EMT) is characterized by the loss of epithelial morphology and the acquisition of mesenchymal characteristics (1). EMT occurs during embryonic development and is required for the formation of tissues and organs. It also plays an important role in pathological processes, such as organ fibrosis and cancer (2,3). The activation of EMT in cancer cells may lead to tumor migration, invasion and dissemination, which are cytological elements that cause cancer metastasis (4).

Transforming growth factor-β1 (TGF-β1) is one of the main EMT-inducing factors in both physiological and pathological conditions (5), by activating EMT-associated transcriptional regulators, such as Snail, Twist and Zinc finger E-box binding homeobox 1 (ZEB1) (6). TGF-β1 exerts profound effects on the growth and EMT-like responses of thyroid epithelial cells, oral squamous cell carcinoma cells and pancreatic cancer cells (7–9). The ability of TGF-β1 to induce EMT plays a critical role in the acquisition of a migratory and invasive phenotype that correlates with an enhanced metastatic potential in tumor cells (26). Epidermal growth factor (EGF) has been shown to disrupt cell-cell junctions and induce EMT in a number of cell lines (10–12). EGF stimulates signaling pathways through the EGF receptor (EGFR), which is associated with a more invasive behavior and a poor prognosis (13,14). TGF-β1 and EGF have been implicated in the process of EMT, and their corresponding intracellular transduction pathways have been reported to form highly interconnected networks; however, the mechanisms involved has not been determined yet (15,16).

Hyaluronan (HA) is an ubiquitous component of the pericellular matrix. Through its interaction with the cell surface receptor, CD44, HA plays key regulatory roles in tissue homeostasis and cancer progression (17). Three isoforms of hyaluronan synthases (HAS) namely HAS1, HAS2 and HAS3, have been identified thus far (18). CD44, an adhesion molecule that binds to HA, has been implicated in cancer cell migration, invasion and metastasis (19). CD44 is composed of a common domain and a variable region of alternatively spliced exons (20). The common domain induces an extracellular region that interacts with its extracellular matrix ligand, HA. CD44 is able to modulate intracellular signaling through the formation of co-receptor complexes with various receptor tyrosine kinases (21). Indeed, increasing evidence points to a role for CD44 as a critical mediator of both growth factor- and HA-induced invasive signaling in cancer cells (22,23).

In the present study, we aimed to investigate the mechanisms through which TGF-β1 induces the EMT process by the transactivation of EGF signaling. The HA-CD44-mediated TGF-β1 and EGF signaling, and the co-localization of CD44/EGFR had an effect on the activation of EGF signaling induced by TGF-β1 in lung and breast cancer cells.

## Materials and methods

### Cell culture and reagents

The MCF-7 breast cancer cell line and the A549 lung adenocarcinoma cell line were obtained from the Type Culture Collection of the Chinese Academy of Sciences, Shanghai, China. The cells were cultured in RPMI-1640 medium supplemented with 10% fetal calf serum (HyClone, Logan, UT, USA) and 100 U/ml of penicillin and 100 mg/l of streptomycin at 37°C in a 5% CO_2_ humidified atmosphere. Logarithmic phase cells were used in the experiments. In some cases, 10% fetal calf serum was changed to serum-free medium depending on the experiment. The cells were treated with EGF at 10 ng/ml or TGF-β1 at 5 ng/ml for 24 h. EGF (AF-100-15) and TGF-β1 (AF-100-18B) were purchased from PeproTech (Rocky Hill, NJ, USA). 4-Methylumbelliferone (4-MU) was obtained from Sigma-Aldrich (St. Louis, MO, USA).

### Western blot analysis

For western blot analysis, the cells were harvested in RIPA lysis buffer. Following incubation at 4°C for 30 min, the lysate was centrifuged at 15,000×g for 10 min at 4°C. The protein concentration was determined using a BCA assay (Pierce, Rorkford, IL, USA). Samples were denatured by 5X sodium dodecyl sulfate-polyacrylamide gel electrophoresis (SDS-PAGE) sample buffer for 5 min at 95°C and subjected to 10% SDS-PAGE. The separated proteins were transferred onto PVDF membranes (Millipore, Bedford, MA, USA) for 2 h at 4°C, blocked in 5% non-fat milk in phosphate-buffered saline/Tween-20 and blotted with antibodies. The following primary antibodies were used: E-cadherin (#3195s), ZEB-1 (#6935s), N-cadherin (#4061s), Snail (#5879s), vimentin (#7391s) (all from Cell Signaling Technology, Beverly, MA, USA), Twist (ab50581; Abcam, Cambridge, UK), EGFR (#4267s), phosphorylated (p-)AKT (#4060s), p-extracellular signal-regulated kinase (ERK; #4370s), p-Smad (#3101s), ERK (#4695), Smad (#5339s) (all from Cell Signaling Technology), β-actin (Sigma-Aldrich), AKT (#2920s; Cell Signaling Technology) and CD44 (sc-18849; Santa Cruz Biotechnology, Inc., Santa Cruz, CA, USA).

### Immunofluorescence staining

For immunofluorescence staining, the cultured cells were fixed for 10 min in 4% paraformaldehyde, permeabilized with 0.1% Triton X-100 and incubated overnight at 4°C with rabbit anti-human EGFR (#4267s; Cell Signaling Technology) and mouse anti-human CD44 (sc-18849; Santa Cruz Biotechnology, Inc.). Subsequently, the sections were rinsed and incubated with the secondary antibody conjugated to the fluorescent dye Alexa Fluor^®^ 488 and Alexa Fluor^®^ 594 (both from Jackson ImmunoResearch, West Grove, PA, USA). The sections were rinsed and incubated with 4′,6-diamidino-2-phenylindole dihydrochloride (DAPI; Life Technologies, Grand Island, NY, USA). A confocal laser scanning microscope (SP5; Leica, Wetzlar, Germany) was used to visualize the immunofluoresence staining.

### RNA isolation, cDNA synthesis and RT-PCR

Total RNA was extracted from the cells using the RNAiso Plus (Takara Bio, Shiga, Japan) according to the manufacturer’s instructions. Reverse transcription was performed with high capacity cDNA reverse transcripting kits (Takara Bio) according to the manufacturer’s instructions. RT-PCR (98°C, 2 min; 36×98°C, 5 sec; 55°C, 30 sec) was carried out using 2X PCR Solution Premix Taq (R004A; Takara Bio). The primer sequences were as follows: TβR, 5′GGCCAAATATCCCAAACAGAT3′ and 5′AATCCAACTCCTTTGCCC3′; HAS1, 5′-GACGTGCGGA TCCTTAACCC-3′ and 5′-CGTTGTACAGCCACTCACG GAA-3′; HAS2, 5′-AAGGCCATTTTCAGAATCCAA-3′ and 5′-TGGCAGAATGAAAATAAACCCAT-3′; HAS3, 5′-CTTTCCTTCATCTCCCACGAAC-3′ and 5′-CAAGCCCT TAGCGAAGTCTG-3′.

### shRNA transfection

shRNA targeting CD44 (shRNA-CD44) (GeneChem, Shanghai, China) was transfected into the cells using Lipofectamine LTX (Life Technologies, Carlsbad, CA, USA) according to the manufacturer’s instructions. The shRNA sequence was CCTCTGCAAGGCTTTCAAT, GCTCTGA GCATCGGATTTG and ATAGCACCTTGCCCACAAT. There were 3 specific shRNAs, the inhibitory effect of which was assessed by western blot analysis (data not shown). Briefly, the most effective shRNA was incubated with PLUS reagent for 5 min, following which, LTX reagent was added. A 30-min incubation at room temperature ensued, and the complex was subsequently applied to the cell culture medium. A negative control shRNA was purchased from GeneChem, the sequence of which was TTCTCCGAACGTGTCACGT.

### Cell migration and invasion assays

Cell migration and invasion assays were conducted using 8 *µ*m Transwell inserts (Corning Inc., Corning, NY, USA) and 24-well BD BioCoat Matrigel Invasion Chambers (BD Biosciences, San Jose, CA, USA) according to the manufacturer’s recommendations. In brief, the cells were seeded into the upper inserts (for migration assay, 5×10^4^ cells/insert were used, and for invasion assay, 1×10^5^ cells/insert were used) with RPMI-1640. The outer wells were filled with RPMI-1640 containing EGF and/or TGF-β1 as the chemoattractant. After 24 h of incubation, the membranes with the migrated or invaded cells were stained with hematoxylin, washed and then mounted on slides. The entire membrane was counted under a light microscope (IX70; Olympus, Tokyo, Japan). Data were expressed as the number of invaded cells per well. Each assay was conducted in triplicate and was repeated at least twice.

### Co-immunoprecipitation (Co-IP) assay

The cells were lysed in ice-cold Co-IP lysis buffer containing 50 mM Tris-HCl (pH 7.4), 150 mM NaCl, 0.5% NP-40, 0.25% Na-deoxycholate, 1 mM EDTA, 50 mM NaF, 1 mM sodium orthovanadate, 0.1 mM PMSF and protease inhibitor cocktail, and were then incubated on ice for 10 min. The insoluble material was pelleted at 13,000×g for 10 min at 4°C. The supernatant was pre-cleaned by protein A/G PLUS-Agarose (Santa Cruz Biotechnology, Inc.) and the aliquots were co-immunoprecipitated with either non-specific IgG or specific antibody against CD44 in Co-IP lysis buffer at 4°C for 1 h, followed by incubation with protein A/G PLUS-Agarose beads for a further 1 h at 4°C. The immunoprecipitated complexes were washed with Co-IP washing buffer [200 mM Tris (pH 7.4), 150 mM NaCl, 0.5% NP-40, and 1 mM EDTA] 5 times and once with Co-IP lysis buffer. The precipitated proteins were then analyzed by western blot analysis. The input was used as a positive control.

### Statistical analysis

Data are presented as the means ± SD and were analyzed by one-way analysis of variance (ANOVA). All the experiments were independently carried out and repeated at least 3 times. A P-value <0.05 was considered to indicate a statistically significant difference.

## Results

### TGF-β1 is an important inducer of EMT compared with EGF

To determine the effects of TGF-β1 and EGF on promoting EMT, we treated the A549 cells with TGF-β1 and EGF at various concentrations (0, 5, 10 and 20 ng/ml) and found the suitable concentrations required for each different cytokine. Subsequently, the cells were exposed to TGF-β1 (5 ng/ml) and EGF (10 ng/ml) for different periods of time (0, 24, 48 and 72 h). The results revealed that TGF-β1 and EGF induced EMT in a dose- and time-dependent manner (data not shown).

**Figure 1:**
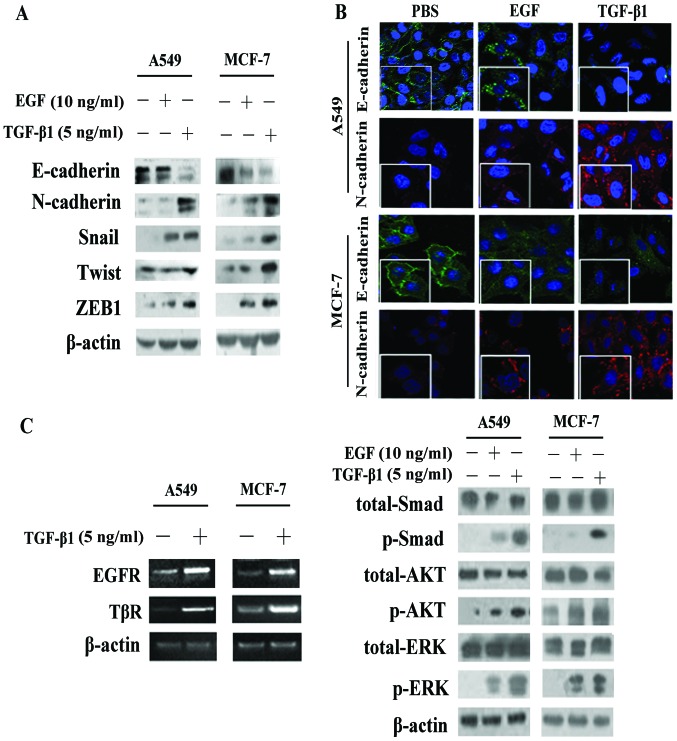
Transforming growth factor-β1 (TGF-β1) is an important inducer of epithelial-mesenchymal transition (EMT) compared with epidermal growth factor (EGF). (A) Western blot analysis of EMT-related proteins in A549 and MCF-7 cells treated with TGF-β1 (5 ng/ml) or EGF (10 ng/ml). (B) Immunofluorescence staining was conducted to detect E-cadherin (green) and N-cadherin (red) expression in the A549 and MCF-7 cells, which were treated with EGF or TGF-β1 for 24 h. The nuclei were visualized with 4′,6-diamidino-2-phenylindole staining (DAPI, blue). Images were taken at ×200 magnification. (C) RT-PCR was used to measure the mRNA levels of EGFR and TGF-β1 receptor (TβR) following stimulation with TGF-β1. Western blot analysis detected the activation of phosphorylated (p-) Smad, downstream of TGF-β1, and the activation of p-AKT and p-ERK, downstream of EGF/EGFR in the A549 and MCF-7 cells following stimulation with TGF-β1 or EGF.

Significant alterations in the expression of EMT-associated proteins (E-cadherin, N-cadherin, Snail, Twist and ZEB1) were observed following stimulation of the cells with TGF-β1 and EGF, although it was clearly evident that TGF-β1 induced EMT to a greater extent, as its effects on the epxression of these proteins were more potent (Fig. 1A and B).

The results of RT-PCR revealed that, in the A549 and MCF-7 cells, the expression levels of both EGFR andTGF-β receptor (TβR) were significantly increased following stimulation with TGF-β1 compared with no stimulation (Fig. 1C). We then separately investigated the activation of pathways following exposure to TGF-β1 or EGF for 4 h. Our data demonstrated that TGF-β1 not only induced the activation of the Smad pathway, but also induced the phosphorylation of AKT and ERK (Fig. 1C), which are commonly considered to be downstream molecules of the EGF/EGFR pathway (13).

**Figure 2:**
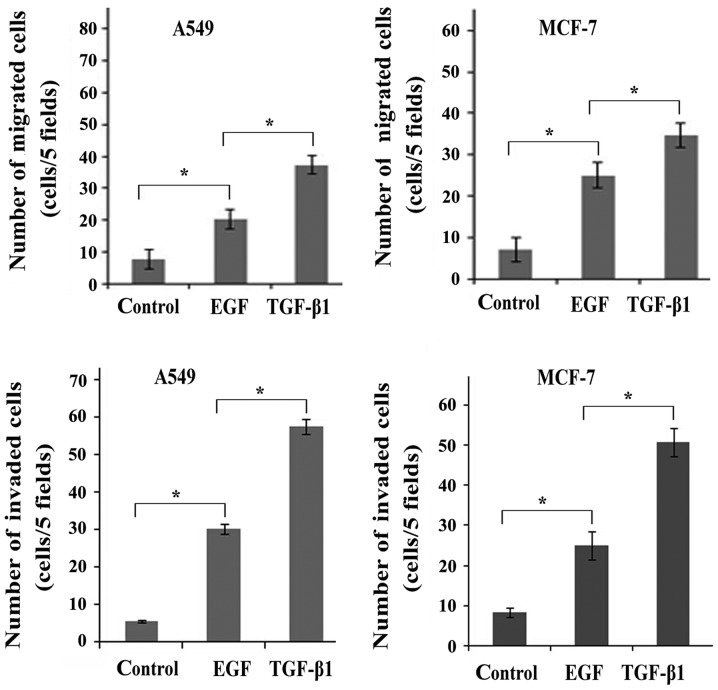
Transforming growth factor-β1 (TGF-β1) is an important inducer of cell migration/invasion compared with epidermal growth factor (EGF). Transwell migration assay revealed the migratory/invasive ability of the cells following stimulation with TGF-β1 (5 ng/ml) or EGF (10 ng/ml). The graphs represent the means ± SD of 3 independent experiments. The y-axis represents the fold change in the number of cells. ^*^P<0.05 vs. control.

The transition between epithelial and mesenchymal cell phenotypes is not only characterized by the expression of EMT markers, but also the biological-functional and behavioral phenotypes (15). As expected, a larger number of both A549 and MCF-7 cells acquired migration and invasion ability following stimulation with TGF-β1 or EGF than the untreated control cells (Fig. 2), suggesting that stimulation with TGF-β1 and EGF led to an enhanced migratory and invasive potential of the cancer cells. In addition, a larger number of migrated/invaded cells was observed following stimulation with TGF-β1 than following stimulation with EGF. Taken together, these findings demonstrated that TGF-β1 induced EMT to a greater extent when compared with EGF. It was also suggested that TGF-β1 transactivates EGF signaling by activating EGFR.

### TGF-β1 induces EGFR and CD44 expression and co-localization

**Figure 3:**
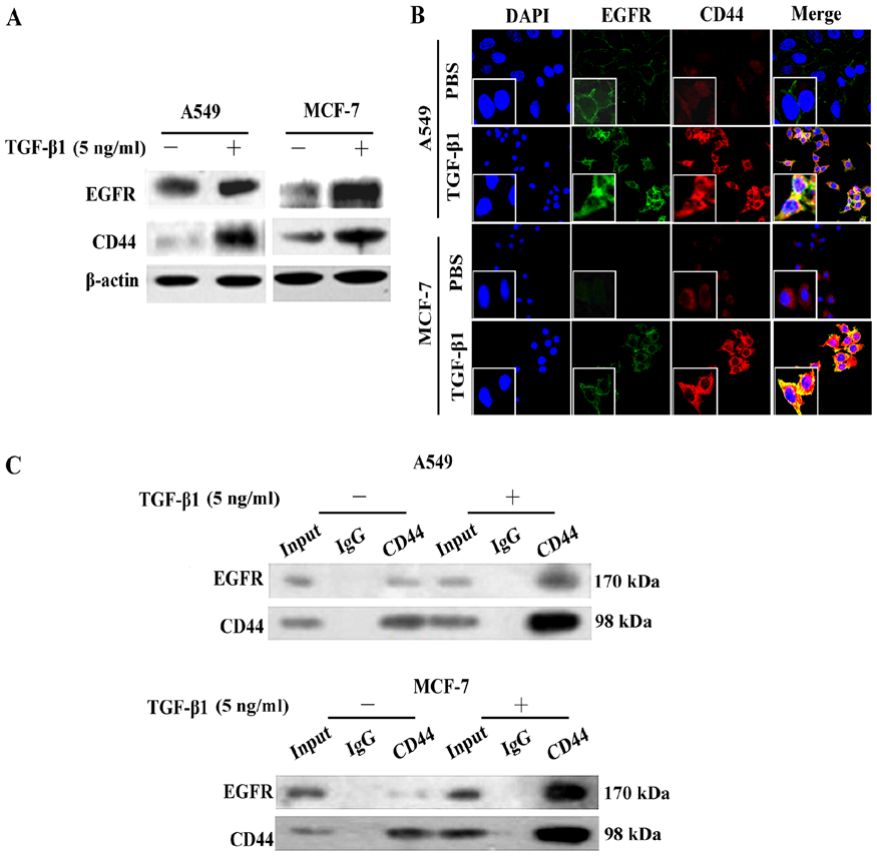
Transforming growth factor-β1 (TGF-β1) induces CD44 and EGFR expression and co-localization. (A) Analysis of the expression levels of epidermal growth factor receptor (EGFR) and CD44 by western blot analysis in A549 and MCF-7 cells, in the absence or presence of TGF-β1. (B) Immunofluorescence staining was conducted to detect EGFR (green) and CD44 (red) expression in A549 and MCF-7 cells, which were stimulated with or without TGF-β1 for 24 h. The nuclei were visualized with 4′,6-diamidino-2-phenylindole staining (DAPI, blue). Images were captured at ×200 magnification. (C) Co-IP analysis was performed using the A549 and MCF-7 cells. EGFR proteins were immunoprecipitated using an antibody against CD44. IgG was used as a negative control.

In accordance with the results of RT-PCR (Fig. 1C), western blot analysis also demonstrated that EGFR cell surface expression levels increased following stimulation of the A549 and MCF-7 cells with TGF-β1 (Fig. 3A). We found that the CD44 expression levels were also increased. Moreover, the expression levels and cellular localization of EGFR and CD44 following stimulation with TGF-β1 were assessed by confocal laser scanning microscopy using the A549 and MCF-7 cells. As shown in Fig. 3B, without TGF-β1 stimulation, the EGFR (green) and CD44 (red) proteins were weakly expressed around the cytomembrane. However, following stimulation with TGF-β1, both the EGFR and CD44 expression levels were upregulated and showed an obvious co-localization, with visible yellow signals (merged image) (Fig. 3B).

In order to explore the role of EGFR/CD44 in TGF-β1-induced EMT and verify the interaction between EGFR and CD44, we performed Co-IP assay. CD44 antibody was used to co-immunoprecipitate EGFR protein from the A549 and MCF-7 cell extracts. Compared with the negative band in the control groups (Fig. 3C, IgG lane) and the weak band in the normal extracts (Fig. 3C, input lane), EGFR protein was successfully immunoprecipitated and was enriched by CD44 antibody (Fig. 3C; represented by a dark band, CD44 lane). These results suggest that TGF-β1 stimulates CD44 and promotes CD44-EGFR complex formation.

### Inhibition of HAS by 4-MU abolishes TGF-β1-induced CD44/EGFR expression and co-localization

**Figure 4:**
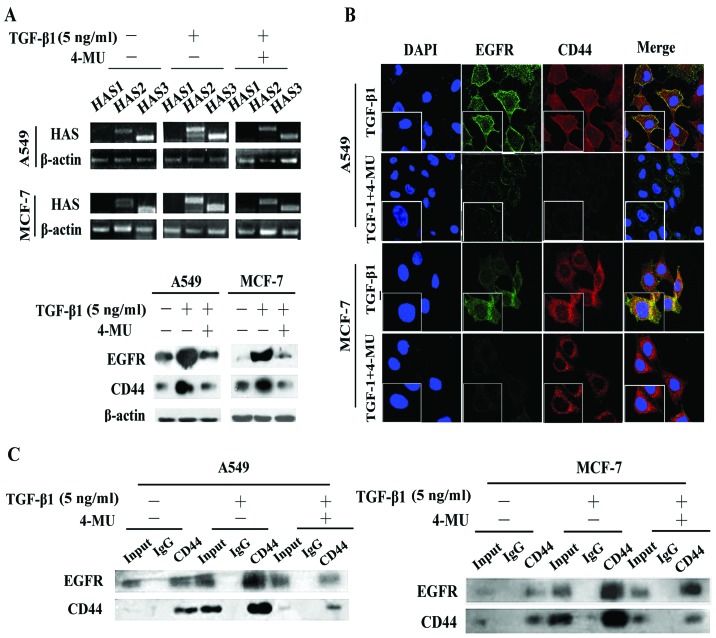
Inhibition of hyaluronan (HA) by 4-methylumbelliferone (4-MU) abolishes the transforming growth factor-β1 (TGF-β1)-induced CD44/epidermal growth factor receptor (EGFR) expression and co-localization. (A) The mRNA expression levels of different HAS subtypes were determined by RT-PCR following stimulation of the A549 and MCF-7 cells with TGF-β1 or TGF-β1 and 4-MU. Changes in mRNA expression levels were then detected. Western blot analysis revealed that CD44 and EGFR protein expression levels were altered following stimulation with TGF-β1 or TGF-β1 plus 4-MU. β-actin was used as a loading control. (B) Immunofluorescence staining was conducted to detect EGFR (green) and CD44 (red) expression in the A549 and MCF-7 cells, that were stimulated with TGF-β1 or TGF-β1 plus 4-MU. The nuclei were visualized with 4′,6-diamidino-2-phenylindole staining (DAPI, blue). Images were captured at ×200 magnification. (C) Co-IP analysis was carried out using the A549 and MCF-7 cells following stimulation with TGF-β1 or TGF-β1 plus 4-MU. EGFR proteins were immunoprecipitated using an antibody against CD44. IgG was used as a negative control.

Hyaluronan synthesis and degradation are regulated by HAS (15). We found that stimulation with TGF-β1 increased both HAS2 and HAS3 expression at the mRNA level (Fig. 4A), suggesting that HAS2 and HAS3 are involved in the TGF-β1-mediated effects on HA production. Moreover, the upregulation of HAS2 was more obvious than that of HAS3 in the A549 and MCF-7 cells, whereas the mRNA expression levels of HAS1 were not altered (Fig. 4A). We then treated the A549 and MCF-7 cells with the HAS inhibitor, 4-MU, and measured the mRNA expression levels of HAS2 and HAS3. The results revealed that the expression levels were significantly downregulated (Fig. 4A). Western blot analysis (Fig. 4A) and immunofluorescence staining (Fig. 4B) also demonstrated that 4-MU suppressed the expression of CD44 and EGFR, decreasing their expression to levels similar to those of the cells not stimulated with TGF-β1 and disrupted the CD44-EGFR co-localization induced by TGF-β1 (Fig. 4A and B). Additionally, 4-MU inhibited the interaction between CD44 and EGFR, even in the presence of TGF-β1 (Fig. 4C).

**Figure 5:**
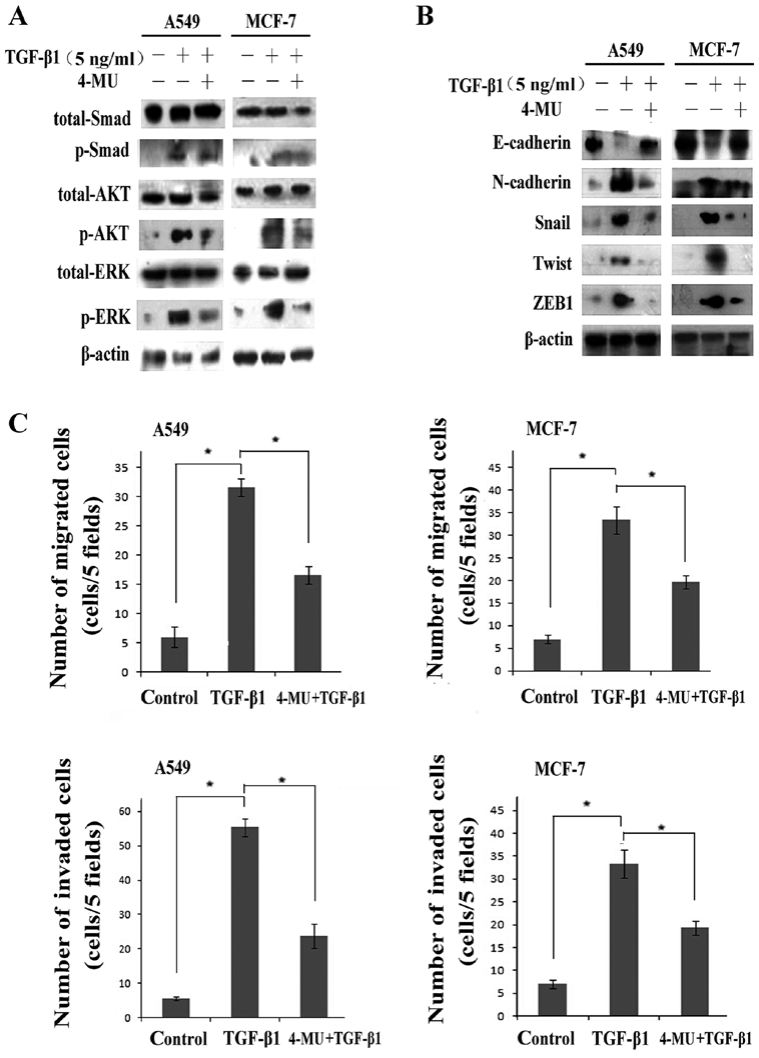
Inhibition of hyaluronan (HA) by 4-methylumbelliferone (4-MU) abolishes the transforming growth factor-β1 (TGF-β1) induced epithelial-mesenchymal transition (EMT). (A and B) Western blot analysis of the expression of downstream pathways and EMT-related proteins, such as E-cadherin, vimentin, Snail and Twist, following stimulation with TGF-β1 or TGF-β1 plus 4-MU. β-actin was used as a loading control. (C) A Transwell assay was carried out to determine the migratory/invasive ability of the cells following stimulation with TGF-β1 and treatment with 4-MU. All graphs represent the means ± SD of 3 independent experiments. The y-axis represents the fold change in the number of cells. ^*^P<0.05 vs. control.

We further investigated the effects of 4-MU on pathway activation. Pathways downstream of EGF/EGFR were shown to be inactivated, the levels of p-ERK and p-AKT were signifi-cantly decreased, whereas the expression levels of total ERK and AKT proteins remained unaltered (Fig. 5A). Moreover, no significant effect on Smad2/3 phosphorylation was observed following treastment with 4-MU, which suggesting suggests that 4-MU suppressed the EGFR pathway rather than the TGF-β1 pathway. In other words, HA contributed to the regulation of TGF-β1-induced EGF/EGFR signaling.

In addition, we detected the expression of a set of EMT markers, including E-cadherin, N-cadherin, Snail, Twist and ZEB1, as well as the cell migration and invasion ability. Western blot assays revealed that the cells treated with 4-MU had higher expression levels of E-cadherin, and lower expression levels of N-cadherin, Snail, Twist and ZEB1 compared with the cells treated with TGF-β1 alone (Fig. 5B), indicating that treatment with 4-MU reversed EMT. Moreover, the results form Transwell migration assay indicated that the number of migrated/invaded cells was markedly decreased following treatment with 4-MU even after stimulation with TGF-β1 (Fig. 5C).

### shRNA-CD44 blocks the activation of the AKT and ERK pathways and causes the reversal of EMT

**Figure 6:**
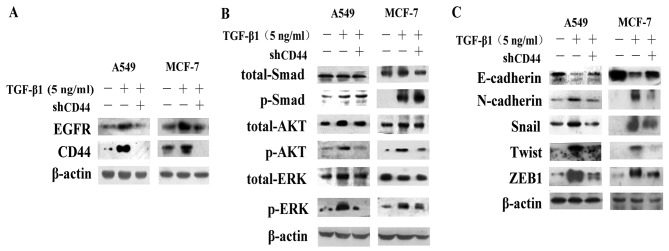
shRNA targeting CD44 (shCD44) blocks the activation of the AKT and ERK pathways and causes the reversal of epithelial-mesenchymal transition (EMT). (A) Western blot analysis revealed that the CD44 protein levels and epidermal growth factor receptor (EGFR) expression were altered following stimulation with transforming growth factor-β1 (TGF-β1) or TGF-β1 plus shRNA. β-actin was used as a loading control. (B and C) Western blot analysis of the expression of downstream pathways and EMT-related proteins, such as E-cadherin, vimentin, Snail and Twist, following stimulation with TGF-β1 or TGF-β1 plus shCD44. β-actin was used as a loading control.

**Figure 7:**
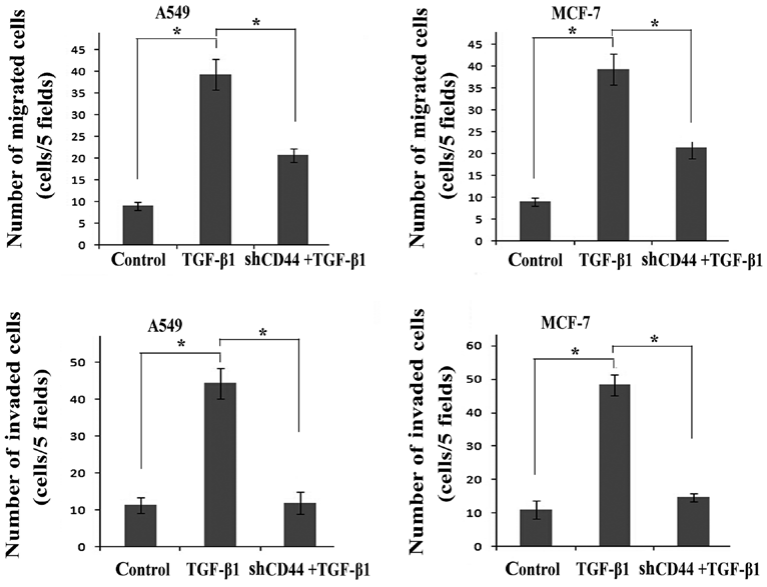
shRNA targeting CD44 (shCD44) blocks the transforming growth factor-β1 (TGF-β1)-induced cell migration/invasion. A Transwell assay was carried out to determine the migratory/invasive ability of the cells following stimulation with TGF-β1 and transfectin with shCD44. All graphs represent the means ± SD of 3 independent experiments. The y-axis represents the fold change in the number of cells. ^*^P<0.05 vs. control.

We then examined whether the disruption of CD44 expression effectively suppresses the expression of EGFR. Transfection of the cells with shRNA-CD44 resulted in a decrease in CD44 and EGFR expression compared to the controls and the TGF-β1-treated group (Fig. 6A). We found that the knockdown of CD44 also caused a marked decrease in the levels of p-ERK and p-AKT, whereas it had no effect on the levels of p-Smad (Fig. 6B), demonstrating the effects of CD44 on TGF-β1-induced EGF/EGFR signaling. Alterations in the expression of EMT-associated proteins indicated that interference with the expression of CD44 reversed EMT (Fig. 6C). Transfection of the cells with shRNA-CD44 increased E-cadherin expression and decreased N-cadherin, Snail, Twist and ZEB1 expression. Cell migration/invasion assays revealed that transfection with shRNA-CD44 partially suppressed cell migration and invasion during the EMT process induced by TGF-β1 (Fig. 7).

In conclusion, the inhibition of CD44 by shRNA-CD44 following stimulation with TGF-β1 abolished the expression of CD44 and EGFR, deactivated the EGF/EGFR signaling pathways and inhibited the process of EMT, suggesting that there is a link between TGF-β1 and CD44/EGFR in the process of EMT.

## Discussion

EMT enables epithelial cells to lose cell-cell adhesions and acquire a mesenchymal phenotype, exhibiting enhanced migratory and tumor-initiating capabilitie. Thus, EMT is considered a crucial factor in cancer metastasis and progression (24). EMT is characterized by a loss of epithelial markers (E-cadherin), while gaining mesenchymal markers (vimentin and N-cadherin) and the upregulation of associated transcriptional regulators (Snail, Twist and ZEB1) (25). The process of EMT involves a variety of regulatory mechanisms supported by diverse extracellular signal-derived activities and gene regulation. Extracellular clues include the extracellular matrix components, TGF-β1 and EGF. TGF-β1 signaling occurs throuhg the phosphorylation of Smad2 and Smad3, and this complex is associated with Smad4 translocation to the nucleus, resulting in the transcriptional activation of downstream targets (26). The EGF/EGFR system regulates numerous biological processes by activating multiple downstream signaling pathways, including the mitogen-activated protein kinase (MEK)-ERK pathway and the phosphoinositide-3 kinase (PI3K)/AKT pathways (27–29).

Kang *et al* (30) demonstrated that the crosstalk between TGF-β1-mediated Smad and EGFR signaling induced trans-membrane 4 L6 family member 5 (TM4SF5) expression and led to the acquisition of mesenchymal cell characteristics (30). Ouyang *et al* (16) reported that miR-10b was engaged in EGF-TGF-β1 crosstalk and enhanced the expression of EMT-promoting genes in pancreatic ductal adenocarcinoma. We observed that either TGF-β1 or EGF alone were able to induce EMT and that stimulation with TGF-β1 was more potent than stimulation with EGF in the regulation of EMT, as measured through a change in the behavioral phenotype and the expression of EMT-associated molecules, further suggesting that TGF-β1 is an important inducer of EMT. Additionally, TGF-β1 increased the expression of EGFR, as well as that of p-AKT and p-ERK, downstream molecules of the EGF/EGFR pathway. There was considerable evidence to support the existence of the transactivation of EGF signaling by TGF-β1 during the process of EMT.

HA, an ubiquitous extracellular and cell surface-associated component of the extracellular matrix, has been shown to negatively correlate with clinical outcomes in several types of cancer, such as breast, colon and prostate cancer (31). HA binds to and signals through the cell surface receptor, CD44, as has been confirmed by previous studies (32,33). The majority of malignant tumors contain elevated levels of both HA and CD44, and it has been demonstrated that HA-activated CD44 promotes the proliferation, invasion, metastasis and stemness of cancer cells (34). Various growth factors and cytokines, such as TGF-β1, have been shown to modulate HA production in tumor cells (17). EGFR acts on CD44 to stimulate Ras-mediated signaling in an HA-dependent manner (35–36). In the present study, we investigated whether HA/CD44 plays an important role in the TGF-β1-induced EGF signaling transactivation and EMT in cancer cells. Our results revealed that TGF-β1 upregulated the expression of HAS2 and HAS3, which increased the expression of CD44, and subsequently promoted CD44/EGFR co-localization, and activated the downstream AKT and ERK pathways. Our results were similar to those of a physiological study, in which the investigators found that the HAS2-dependent production of HA facilitates TGF-β1-dependent fibroblast differentiation by promoting the interaction between CD44 and EGFR held within membrane-bound lipid rafts (37).

In this study, to determine the importance of HA/CD44 in TGF-β1-induced EGF signaling activation and EMT in cancer cells, we used the HAS inhibitor, 4-MU, to block HAS expression and shRNA targeting CD44 to knockdown CD44 expression. We observed that they both 4-MU and shRNA-CD44 abolished TGF-β1-induced CD44/EGFR expression. Co-IP and immunofluorescence staining revealed that the inhibition of HAS disrupted CD44 and EGFR co-localization. In addition, the downregulation of HAS and CD44 blocked the TGF-β1-induced activation of the EGFR pathway, presented as a decrease in the phosphorylation levels of AKT and ERK, and reversed EMT. This indicates that HA/CD44 mediates EGF signaling transactivation by TGF-β1 and is vital to TGF-β1-induced EMT. Moreover, we obtained consistent results from two types of cancer cells, suggesting that this mechanism may be common to different types of cancer.

In conclusion, the findings of the current study demonstrated that stimulation with TGF-β1 was more potent than EGF in regulating EMT-associated protein expression and enhancing cell migration and invasion. More importantly, the data presented in this study indicated that TGF-β1 upregulated HAS expression, promoted CD44/EGFR expression and co-localization, and subsequently activated the AKT and ERK signaling pathways. Furthermore, the inhibition of HAS by 4-MU and that of CD44 by shRNA abolished TGF-β1-induced CD44/EGFR expression, inhibited the activation of the AKT and ERK pathways and caused the reversal of EMT. The aforementioned results suggest that HA/CD44 contributes to the transactivation of EGF signaling through TGF-β1 in EMT during cancer progression. Studies designed to elucidate the effects of TGF-β1-induced HA/CD44 expression on other EMT-associated signaling pathways, such as platelet-derived growth factor (PDGF)/signal transducers and activators of transcription (STATs) are currently in progress. The detailed analysis of HA/CD44 is important for the effective targeting of EMT and may offer new options for novel anticancer therapeutic strategies.

## References

[1] Wang Y, Shang Y (2013). Epigenetic control of epithelial-to-mesenchymal transition and cancer metastasis. Exp Cell Res.

[2] Frisch SM, Schaller M, Cieply B (2013). Mechanisms that link the oncogenic epithelial-mesenchymal transition to suppression of anoikis. J Cell Sci.

[3] Peng Z, Wang CX, Fang EH, Wang GB, Tong Q (2014). Role of epithelial-mesenchymal transition in gastric cancer initiation and progression. World J Gastroenterol.

[4] Quan J, Elhousiny M, Johnson NW, Gao J (2013). Transforming growth factor-β1 treatment of oral cancer induces epithelial-mesenchymal transition and promotes bone invasion via enhanced activity of osteoclasts. Clin Exp Metastasis.

[5] Sengupta S, Jana S, Biswas S, Mandal PK, Bhattacharyya A (2013). Cooperative involvement of NFAT and SnoN mediates transforming growth factor-β (TGF-β) induced EMT in metastatic breast cancer (MDA-MB 231) cells. Clin Exp Metastasis.

[6] Argast GM, Krueger JS, Thomson S, Sujka-Kwok I, Carey K, Silva S, O’Connor M, Mercado P, Mulford IJ, Young GD (2011). Inducible expression of TGFβ, snail and Zeb1 recapitulates EMT in vitro and in vivo in a NSCLC model. Clin Exp Metastasis.

[7] Toda S, Matsumura S, Fujitani N, Nishimura T, Yonemitsu N, Sugihara H (1997). Transforming growth factor-β1 induces a mesenchyme-like cell shape without epithelial polarization in thyrocytes and inhibits thyroid folliculogenesis in collagen gel culture. Endocrinology.

[8] Richter P, Umbreit C, Franz M, Berndt A, Grimm S, Uecker A, Böhmer FD, Kosmehl H, Berndt A (2011). EGF/TGFβ1 co-stimulation of oral squamous cell carcinoma cells causes an epithelial-mesenchymal transition cell phenotype expressing laminin 332. J Oral Pathol Med.

[9] Liu Z, Du R, Long J, Dong A, Fan J, Guo K, Xu Y (2012). JDP2 inhibits the epithelial-to-mesenchymal transition in pancreatic cancer BxPC3 cells. Tumour Biol.

[10] Chen J, Wang T, Zhou YC, Gao F, Zhang ZH, Xu H, Wang SL, Shen LZ (2014). Aquaporin 3 promotes epithelial-mesenchymal transition in gastric cancer. J Exp Clin Cancer Res.

[11] Li L, Han R, Xiao H, Lin C, Wang Y, Liu H, Li K, Chen H, Sun F, Yang Z (2014). Metformin sensitizes EGFR-TKI-resistant human lung cancer cells in vitro and in vivo through inhibition of IL-6 signaling and EMT reversal. Clin Cancer Res.

[12] Davis FM, Peters AA, Grice DM, Cabot PJ, Parat MO, Roberts-Thomson SJ, Monteith GR (2012). Non-stimulated, agonist-stimulated and store-operated Ca2+ influx in MDA-MB-468 breast cancer cells and the effect of EGF-induced EMT on calcium entry. PLoS One.

[13] Berndt A, Büttner R, Gühne S, Gleinig A, Richter P, Chen Y, Franz M, Liebmann C (2014). Effects of activated fibroblasts on phenotype modulation, EGFR signalling and cell cycle regulation in OSCC cells. Exp Cell Res.

[14] Ren S, Su C, Wang Z (2014). Epithelial phenotype as a predictive marker for response to EGFR-TKIs in non-small cell lung cancer patients with wild-type EGFR. Int J Cancer.

[15] Tian YC, Chen YC, Chang CT, Hung CC, Wu MS, Phillips A, Yang CW (2007). Epidermal growth factor and transforming growth factor-beta1 enhance HK-2 cell migration through a synergistic increase of matrix metalloproteinase and sustained activation of ERK signaling pathway. Exp Cell Res.

[16] Ouyang H, Gore J, Deitz S, Korc M (2014). microRNA-10b enhances pancreatic cancer cell invasion by suppressing TIP30 expression and promoting EGF and TGF-β actions. Oncogene.

[17] Chow G, Tauler J, Mulshine JL (2010). Cytokines and growth factors stimulate hyaluronan production: role of hyaluronan in epithelial to mesenchymal-like transition in non-small cell lung cancer. J Biomed Biotechnol.

[18] Porsch H, Bernert B, Mehić M, Theocharis AD, Heldin CH, Heldin P (2013). Efficient TGFβ-induced epithelial-mesenchymal transition depends on hyaluronan synthase HAS2. Oncogene.

[19] Hiscox S, Baruha B, Smith C, Bellerby R, Goddard L, Jordan N, Poghosyan Z, Nicholson RI, Barrett-Lee P, Gee J (2012). Overexpression of CD44 accompanies acquired tamoxifen resistance in MCF7 cells and augments their sensitivity to the stromal factors, heregulin and hyaluronan. BMC Cancer.

[20] Goodison S, Urquidi V, Tarin D (1999). CD44 cell adhesion molecules. Mol Pathol.

[21] Hiraga T, Ito S, Nakamura H (2013). Cancer stem-like cell marker CD44 promotes bone metastases by enhancing tumorigenicity, cell motility, and hyaluronan production. Cancer Res.

[22] Midgley AC, Bowen T, Phillips AO, Steadman R (2013). MicroRNA-7 inhibition rescues age-associated loss of EGF receptor and hyaluronan (HA)-dependent differentiation in fibroblasts. Aging Cell.

[23] Williams K, Motiani K, Giridhar PV, Kasper S (2013). CD44 integrates signaling in normal stem cell, cancer stem cell and (pre) metastatic niches. Exp Biol Med (Maywood).

[24] Xu Z, Jiang Y, Steed H, Davidge S, Fu Y (2010). TGFβ and EGF synergistically induce a more invasive phenotype of epithelial ovarian cancer cells. Biochem Biophys Res Commun.

[25] Wendt MK, Smith JA, Schiemann WP (2010). Transforming growth factor-β-induced epithelial-mesenchymal transition facilitates epidermal growth factor-dependent breast cancer progression. Oncogene.

[26] Ohshio Y, Teramoto K, Hashimoto M, Kitamura S, Hanaoka J, Kontani K (2013). Inhibition of transforming growth factor-β release from tumor cells reduces their motility associated with epithelial-mesenchymal transition. Oncol Rep.

[27] Elloul S, Kedrin D, Knoblauch NW, Beck AH, Toker A (2014). The adherens junction protein afadin is an AKT substrate that regulates breast cancer cell migration. Mol Cancer Res.

[28] Voon DC, Wang H, Koo JK, Chai JH, Hor YT, Tan TZ, Chu YS, Mori S, Ito Y (2013). EMT-induced stemness and tumorigenicity are fueled by the EGFR/Ras pathway. PLoS One.

[29] Buonato JM, Lazzara MJ (2014). ERK1/2 blockade prevents epithelial-mesenchymal transition in lung cancer cells and promotes their sensitivity to EGFR inhibition. Cancer Res.

[30] Kang M, Choi S, Jeong SJ, Lee SA, Kwak TK, Kim H, Jung O, Lee MS, Ko Y, Ryu J (2012). Cross-talk between TGFβ1 and EGFR signalling pathways induces TM4SF5 expression and epithelial-mesenchymal transition. Biochem J.

[31] Heffler M, Golubovskaya VM, Conroy J, Liu S, Wang D, Cance WG, Dunn KB (2013). FAK and HAS inhibition synergistically decrease colon cancer cell viability and affect expression of critical genes. Anticancer Agents Med Chem.

[32] Su CY, Li YS, Han Y, Zhou SJ, Liu ZD (2014). Correlation between expression of cell adhesion molecules CD44 v6 and E-cadherin and lymphatic metastasis in non- small cell lung cancer. Asian Pac J Cancer Prev.

[33] Cheng C, Yaffe MB, Sharp PA (2006). A positive feedback loop couples Ras activation and CD44 alternative splicing. Genes Dev.

[34] Raso-Barnett L, Banky B, Barbai T, Becsagh P, Timar J, Raso E (2013). Demonstration of a melanoma-specific CD44 alternative splicing pattern that remains qualitatively stable, but shows quantitative changes during tumour progression. PLoS One.

[35] Perez A, Neskey DM, Wen J, Pereira L, Reategui EP, Goodwin WJ, Carraway KL, Franzmann EJ (2013). CD44 interacts with EGFR and promotes head and neck squamous cell carcinoma initiation and progression. Oral Oncol.

[36] Grass GD, Tolliver LB, Bratoeva M, Toole BP (2013). CD147, CD44, and the epidermal growth factor receptor (EGFR) signaling pathway cooperate to regulate breast epithelial cell invasiveness. J Biol Chem.

[37] Midgley AC, Rogers M, Hallett MB, Clayton A, Bowen T, Phillips AO, Steadman R (2013). Transforming growth factor-β1 (TGF-β1)-stimulated fibroblast to myofibroblast differentiation is mediated by hyaluronan (HA)-facilitated epidermal growth factor receptor (EGFR) and CD44 co-localization in lipid rafts. J Biol Chem.

